# A geographical perspective on the relationship between *Impatiens* spur lengths and bill lengths of sunbirds in Afrotropical mountains

**DOI:** 10.1002/ece3.7258

**Published:** 2021-02-10

**Authors:** David Hořák, Štěpán Janeček

**Affiliations:** ^1^ Department of Ecology Faculty of Science Charles University in Prague Praha 2 Czech Republic

**Keywords:** Africa, forest undergrowth, nectarivory, plant–bird interaction, pollination

## Abstract

Trait matching—a correlation between the morphology of plants and their pollinators—has been frequently observed in pollination interactions. Different intensities of natural selection in individual regions should cause such correlations to be observable across different local assemblages. In this study, we focused on matching between spur lengths of the genus *Impatiens* and bill lengths of sunbirds in tropical Africa. For 25 mountain and island locations, we compiled information about the composition and traits of local *Impatiens* and sunbird assemblages. We found that assemblage mean and maximum values of bill lengths were positively correlated with mean and maximum spur lengths across locations. Moreover, our results suggest that the positive correlations hold only for forest sunbird assemblages sharing the same habitat with *Impatiens* species. We further show that long‐billed sunbirds seem to locally match the morphology of multiple *Impatiens* plant species, not vice versa. Our observation implies that trait matching significantly contributes to structuring of *Impatiens–*sunbird pollination systems. We suggest that special habitat preferences together with spatial isolation of mountain environment might play a role in this case.

## Graphical abstract

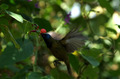

## 1 INTRODUCTION

The importance of specific morphological adaptations in pollination interactions between plants and birds has been a focus of researchers for a long time (Janeček et al., 2012; Snow & Snow, 1972, 1980; Snow & Teixeira, 1982). In both taxonomical groups, morphological traits related to their mutual interaction evolved independently multiple times, as they have been documented across phylogenetic lineages, which makes them a popular example of convergent evolution (Cronk & Ojeda, 2008).

In plants, the most specialized ornithophilous species have tubular flowers that offer nectar especially to the often narrow guild of long‐billed specialist nectar‐feeding birds (Maruyama et al., 2014; Rebelo, 1987; Stiles, 1981). These species are highly adapted and enter specific co‐evolutionary relationships (Abrahamczyk et al., 2014; Stiles, 1981). Longer flowers increase the effectiveness of the pollination process by forcing birds to put their heads deeper into the flower (Temeles et al., 2013; but see Missagia & Alves, 2018) or by excluding short‐billed members of the pollinator assemblage (Cronk & Ojeda, 2008).

Bill length is a classical example of a trait associated with nectarivory in birds (Paton & Collins, 1989). Although birds can consume nectar even from longer flowers using their protruding tongue, this behavior markedly increases handling time. As a result, birds with short bills aim to pick flowers with short corollas (Collins, 2008; Montgomerie, 1984; Paton & Collins, 1989). On the other hand, birds with long bills can drink effectively from both long and short flowers (Montgomerie, 1984). Still, under natural conditions, they often select longer flowers because they produce higher amounts of nectar that cannot be easily extracted by short‐billed birds (Geerts & Pauw, 2009; Janeček et al., 2011; Kaczorowski et al., 2005).

Consequently, in natural communities, avian bill lengths and corolla lengths of visited plants are usually positively correlated. Such trait matching between bills of nectarivorous birds and flowers of ornithophilous plants is predicted to underlay hypotheses on co‐evolutionary processes between these two groups of organisms (Brown et al., 2011; Dalsgaard et al., 2009; del Coro Arizmendi & Ornelas, 1990; Janeček et al., 2011; Nattero & Cocucci, 2007).

Nevertheless, differences in the degree of morphological specialization can be observed not only between individual species within plant and bird communities but also between communities at different geographical scales. It has, for example, been shown that New World birds and their food plants tend to be more specialized compared to their Old World counterparts (Fleming & Muchhala, 2008) and that two main groups of hummingbirds (hermits and nonhermits), which differ in bill size and geographical distribution, utilize taxonomically and ecologically different plant species with corresponding flower morphology (Stiles, 1981, but see Gonzalez & Loiselle, 2016). Such observations imply that interspecific trait matching can reflect in overall characteristics of the whole communities, and thus, a correlation between trait values of interacting plants and birds can be observed among communities with different (intensities of) selection pressures. A phenotypic correlation at such a coarse scale is likely to be connected to the presence of highly specialized guilds of birds and plants in some communities and their absence in others, as has been shown in South America (Buzato et al., 2000; Maruyama et al., 2014). In contrast, it has been proven difficult to find such an intercommunity correlation in Australia, a continent with relatively little specialized plant and bird species (Franklin & Noske, 2000), but see Biddick and Burns (2018) conclusions from the New Zealand plant–bird pollination networks.

Ideas on trait matching between birds and plants in Africa stem mainly from studies performed in subtropical southern parts of the region. Many phylogenetic lineages of plants occurring there have been documented to be adapted to pollination by birds (e.g., Ericaceae and Proteaceae). On the contrary, the number of specialized nectarivorous birds (which includes mainly sunbirds and sugarbirds) is rather low in South Africa (Rebelo, 1987). Still, examples of trait matching in pollination systems have been observed there. For instance, Geerts and Pauw (2009) demonstrated that the long‐billed Malachite Sunbird (*Nectarinia famosa*) of the Cape region is significantly related to a subset of plants with long floral tubes. However, due to environmental differences, information obtained in South Africa cannot be uncritically extrapolated to the whole continent, and unfortunately, almost no data are available from tropical parts of Africa, where most of sunbird diversity can be observed (Cheke et al., 2001). Besides, pollination interactions likely affect geographical distributions of the species (Phillips et al., 2020) and large‐scale studies on pollination systems are rare (but see Zanata et al., 2017). Therefore, at the scale of the continent, we need more insight into the matching of morphological traits between birds and plants involved in pollination interactions. Such an insight can allow us to construct, in the future also to test, related evolutionary and biogeographical hypotheses.

**FIGURE 1:**
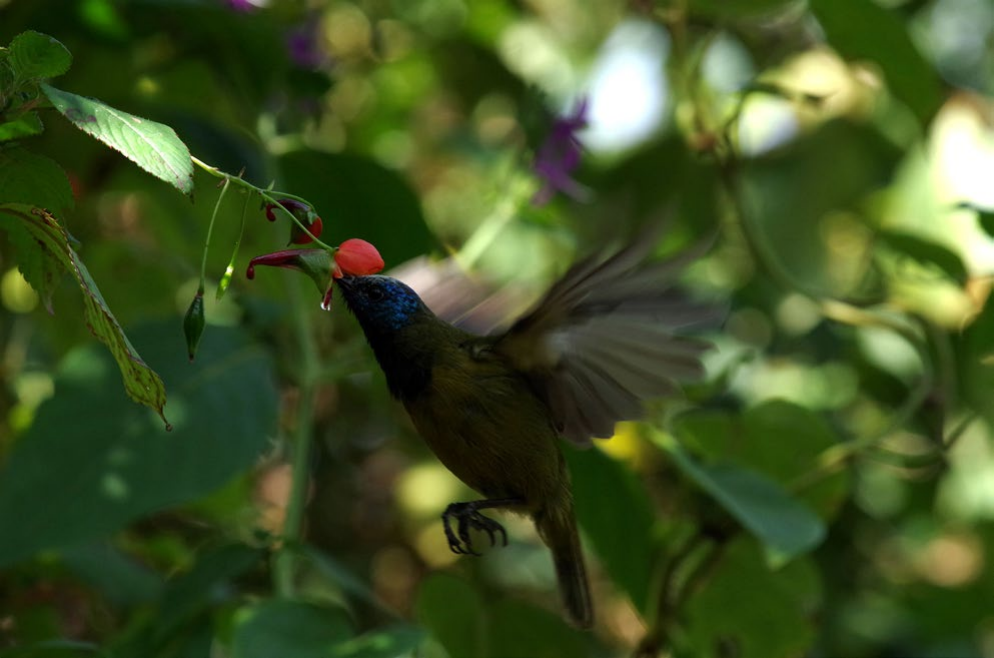
The Cameroon Sunbird (*Cyanomitra oritis*) feeding on *Impatiens sakeriana*. © Štěpán Janeček

Among Afrotropical plants, only a few ornithophilous species have been investigated. Some lobelias seem to be specialized such as *Lobelia telekii* (Evans, 1996), while other (e.g., *Lobelia keniensis* and *Lobelia deckenii*) are pollinated by a wider spectrum of birds (Burd, 1995; Young, 1982). *Leonotis nepetifolia* is pollinated by large sunbirds, but its flower shape does not show specific adaptations to particular species (Gill & Conway, 1979). Members of the Loranthaceae family are well known to be specialized for bird pollination (Polhill & Wiens, 1998), but again no adaptations for particular bird species have been reported (Gill & Wolf, 1975; Weston et al., 2012). Tight specializations have been suggested within the families Marantaceae and Balsaminaceae. Marantaceae species are visited exclusively by long‐billed *Cyanomitra olivacea* (Ley & Classen‐Bockhoff, 2009), and *Impatiens sakeriana* is highly specialized for the long‐billed species *Cyanomitra oritis* (Figure 1; Janeček et al., 2011). Within the plant community, *Impatiens sakeriana* is the species most specialized for ornithophily and produces the highest amounts of nectar (Bartoš et al., 2012). Moreover, our recent research show that five species, bearing the bird pollination syndrome, are also pollinated by long‐billed *Cyanomitra oritis* on Mt. Cameroon and represent the crucial nectar source in the wet season (Bartoš & Janeček, 2014; Janeček et al., 2015). Although our current knowledge of the *Impatiens*–sunbird pollination system is based exclusively on studies from the Cameroon Mountains, *Impatiens* species seem to represent highly morphologically and ecologically specialized plants playing an important role in plant–bird interactions in montane forests throughout tropical Africa (Grey‐Wilson, 1980; Janeček et al., 2012).

Geographical isolation of individual mountains and their eco‐climatic stability during Pliocene and Pleistocene climatic oscillations have contributed to the extraordinary high number of frequently restricted range species of sunbirds and ornithophilous *Impatiens* (Fjeldsa & Lovett, 1997; Janssens et al., 2009). Moreover, the spatial structure of montane environments resembling archipelagos offers a unique opportunity to treat individual mountains or mountain ranges as different units of analysis within which local communities evolved partly independently for a certain amount of time.

In this study, we focused on *Impatiens*–sunbird pollination systems over a large spatial scale in tropical Africa. By relating two morphological traits likely associated with pollination/nectarivory adaptations (spur lengths of plants and bill lengths of birds) among different montane communities, we searched for a large‐scale phenotype correlation which would suggest the importance of trait matching in structuring of *Impatiens* and sunbird communities in the Afrotropical montane environment. Specifically, we tested three main hypotheses: (a) spur lengths of *Impatiens* species are positively correlated with bill lengths of long‐billed sunbird species among different assemblages, which is a result of strong interaction between taxa suggested by previous studies (Bartoš & Janeček, 2014; Bartoš et al., 2012; Janeček et al., 2011, 2012, 2015); (b) Spurs of *Impatiens* that live in the forest match the bills of forest sunbird species, but do not match the bills of nonforest sunbirds occurring on the same mountains. We expect this as habitat preferences of nonforest sunbirds basically limit the probability of interaction with *Impatiens* plants. (c) In addition, we tested for specialization asymmetry in the *Impatiens*–sunbird pollination system, that is, the situation when there is trait matching between more *Impatiens* species and one (or a few) sunbird species. This expectation arises from our empirical studies on Mt. Cameroon where we observed that just small subset of sunbirds interacts with all ornithophilous *Impatiens* spp. (Janeček et al., 2015).

## 2 METHODS

**FIGURE 2:**
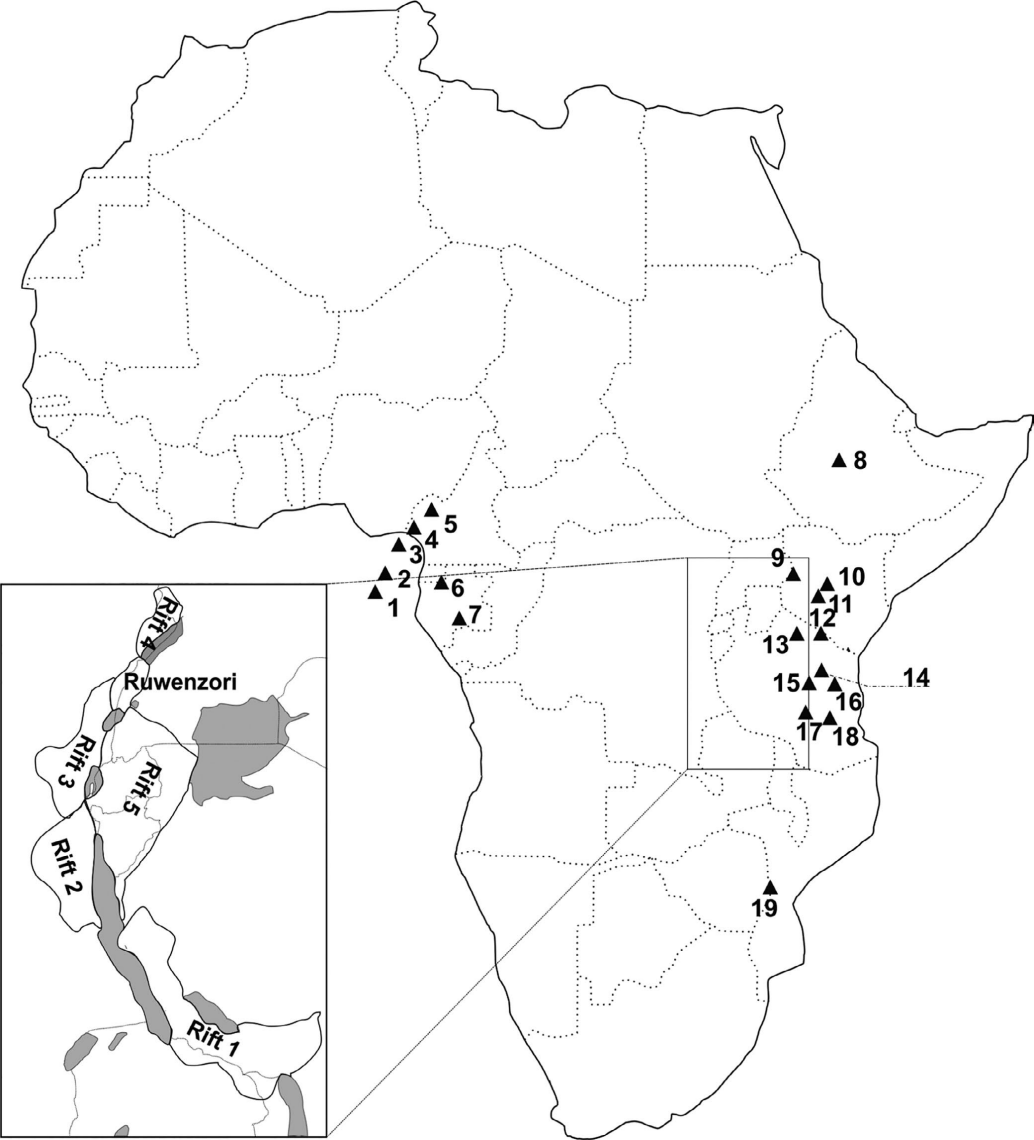
Target mountains and islands: 1/Sao Tomé; 2/Principe; 3/Bioko; 4/Mt. Cameroon; 5/Cameroon Mts.; 6/Crystal Mts.; 7/Massif du Chaillu; 8/Ethiopian Highlands; 9/Mt. Elgon; 10/Mt. Kenya; 11/Aberdare Mts.; 12/Mt. Kilimanjaro; 13/Ngorongoro; 14/Nguru Mts.; 15/Ukaguru Mts.; 16/Uluguru Mts.; 17/Udzungwa Mts.; 18/Mahange; 19/Chimanimani Mts

For the purpose of this study, we selected 25 locations situated within the tropical zone of West‐Central and Eastern Africa (Figure 2 and Table S1). We chose the study locations to cover the most important mountains or mountain areas in sub‐Saharan Africa from which occurrence of *Impatiens* plants and sunbirds have been reported. For each location, we recorded the presence/absence of particular sunbird taxa based on information in the Birds of Africa handbook (Fry et al., 2000). In addition, using the information provided by Fry et al. (2000), we classified birds as forest and nonforest species. We combined distribution maps with information in the text to describe the geographical distribution of sunbirds across selected locations. We employed the same approach to map the distribution of *Impatiens* plants. We extracted plant data from Grey‐Wilson (1980), Cheek and Fischer (1999), Cheek and Csiba (2002), Fischer et al. (2003), Pócs (2007), and Janssens et al. (2010) and from herbarium specimens available on the Global Plants website (http://plants.jstor.org).

### 2.1 Bird morphology

We collected information about bill lengths of sunbirds from the Birds of Africa handbook (Fry et al., 2000). We used or calculated average values of bill length for particular subspecies of sunbirds. If information about a subspecies of interest was not available, we used the mean value for the species instead.

### 2.2 Plant morphology

We considered a species with bright red or orange flowers as ornithophilous (Grey‐Wilson, 1980), which was supported by our previous studies (Janeček et al., 2011, 2012) and our unpublished field observations. *Impatiens* spur lengths were measured on digitalized specimens downloaded from the Global Plants website (http://plants.jstor.org). When specimens were not available, we used the information from *Impatiens* of Africa (Grey‐Wilson, 1980) or for more recently described species from individual taxonomical studies (Cheek & Csiba, 2002; Cheek & Fischer, 1999; Fischer et al., 2003; Janssens et al., 2010; Pócs, 2007). We estimated length of the spur as a distance between the upper point of the spur entrance to the point where the spur bends down and usually back (most of the species) or it is divided into more very narrow spur parts (*I. tricaudata*, *I. digitata*). The only exception was *I. hians* with extremely wide entrance where according to our observations the bill is inserted from the approximately middle of the entrance; in this case, we used middle point of the entrance.

### 2.3 Statistical analyses

Units of statistical analyses were local avian and plant assemblages at selected locations (see Figure 2). For each location, we calculated a mean value for bill length of sunbirds and a mean value of spur length for *Impatiens* flowers. The mean values of traits provide overall information about morphological adaptation of the focal assemblage. By using these values, we were able to test for morphological trait matching between plants and birds among locations. However, a tight morphological matching likely occurs only among some of the community members. To provide a deeper insight into the functional structure of local assemblages, we decided to also include information about maximum and minimum bill and spur lengths. While maximum values give us information about the most intensive selection, as long bills as well as long spurs are thought to be signs of high degree of specialization, there is no such prediction about minimum bill and spur lengths within communities.

The differences between bill lengths of forest and nonforest sunbird species were tested by one‐way ANOVA. To explore relationship between *Impatiens* spur and sunbird bill lengths, we used correlation analyses (Pearson's *r*). However, as we used geographical locations, we corrected the *p*‐values of the correlations for spatial structure in the data. The spatial correlation we employed uses information about geographical coordinates to correct degrees of freedom, we did these correlations in SAM v4.0 software (Rangel et al., 2010). This correction should control for nonindependence in the data due to spatial proximity. We present group data as mean ± *SE*. We performed data processing and all statistical analyses in R 3.0.2 (R Development Core Team, 2013).

## 3 RESULTS

In total, we collected morphological information about 37 taxonomical units of the genus *Impatiens* and about 108 species/subspecies of sunbirds. On average, the spur length of *Impatiens* flowers was 19.18 ± 0.88 mm, and the mean bill length of sunbirds was 22.13 ± 0.53 mm. We did not find any differences between average bill length of forest (22.16 ± 0.84 mm, *n* = 51) and nonforest species (22.11 ± 0.68, *n* = 57); one‐way ANOVA: *F*_(1, 106)_ = 0.002, *p* = .961.

**FIGURE 3:**
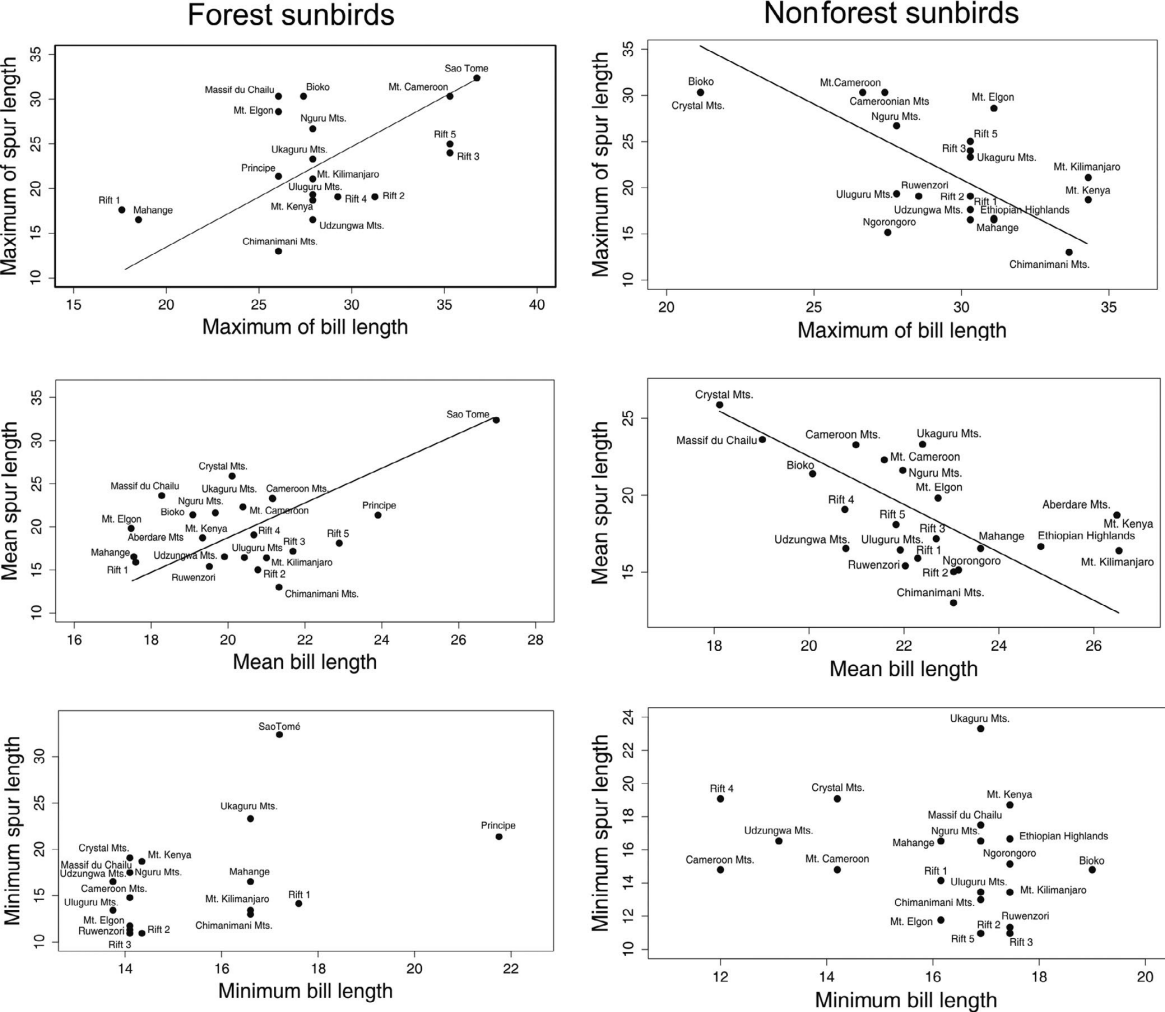
Relationships between spur lengths in *Impatiens* and bill lengths in Sunbird assemblages. Maximum, mean, and minimum values are compared separately for forest and nonforest sunbird assemblages

We analyzed the relationship between spur length of *Impatiens* species and bill length separately for forest and nonforest species of sunbirds (Figure 3). For forest species, we found maximum bill length to be positively correlated with maximum spur length within the focal assemblages (Table 1). Similarly, average values of bill lengths were significantly and positively related to average values of spur lengths (Table 1). However, we found a marginally nonsignificant relationship between minimum values of bill length and minimum values of spur length (Table 1).

**TABLE 1:** The correlations between *Impatiens* spur lengths and bill lengths of sunbirds in individual communities

	*n*	*r*	*p*	*p*_sc_
Forest sunbird species				
Maximal lengths	19	.489	.029	.054
Average lengths	23	.4456	.030	.039
Minimal lengths	17	.434	.073	.108
Nonforest sunbird species				
Maximal lengths	20	−.613	.003	.080
Average lengths	23	−.522	.009	.058
Minimal lengths	21	−.253	.257	.271

Note

We considered either sunbird forest or nonforest communities.

Abbreviation: *p*_sc_, spatially corrected *p* value.

For nonforest species of sunbirds, we found no positive correlation between spur lengths of *Impatiens* species and bill lengths of sunbirds. Maximum length of the spur was negatively related to maximum bill length (Table 1). We revealed a similar trend between average values of spur and bill lengths (Table 1). The minimum values of bill and spur length showed a nonsignificant correlation (Table 1) among the focal assemblages.

**FIGURE 4:**
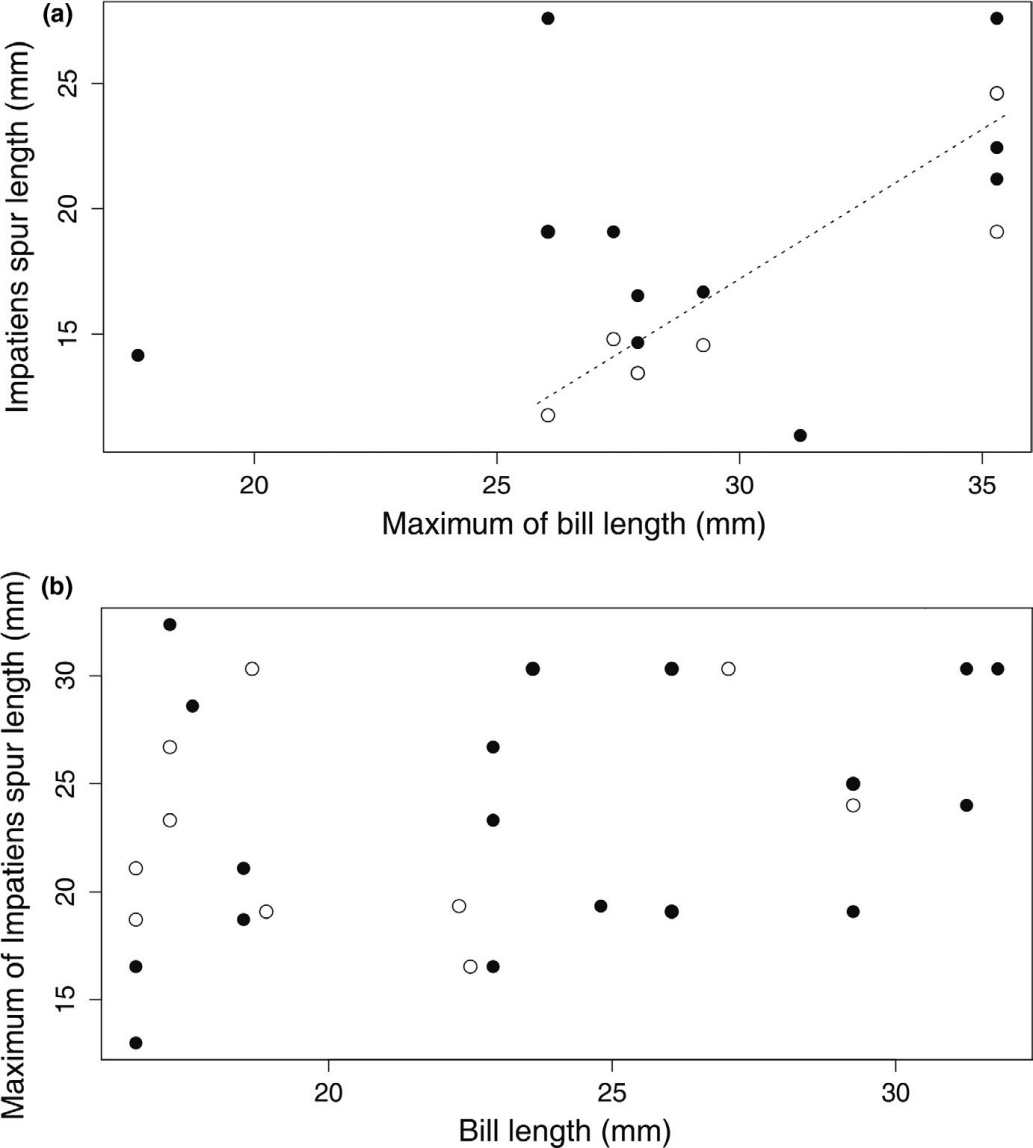
(a) Relationships between maximum bill length values of sunbirds and (i) the second longest spur (filled circle; spatially corrected *p* = .184), (ii) the third longest spur (open circle, dashed line; spatially corrected *p* = .007) in *Impatiens* assemblages. (b) Relationships between maximum spur lengths in *Impatiens* plants and (i) the second longest bill (filled circle; spatially corrected *p* = .216), (ii) the third longest bill (open circle; spatially corrected *p* = .68) in the sunbird assemblages

To explore the possible specialization asymmetry, we decided to compare maximum values of bill and spur length with lower‐order values for these two traits for particular local assemblages (Figure 4). We found a positive trend between maximum bill length and the second longest spur length; however, the relationship was nonsignificant (*n* = 11, *r* = .386, *p* = .219, spatially corrected *p* = .184). Further, we found a positive and significant correlation between maximum bill size and the third longest spur length within assemblages (*n* = 9, *r* = .806, *p* = .006, spatially corrected *p* = .007). In addition, we tested for relationships between the maximum spur length and lower‐order values for bill lengths within assemblages. We found no significant correlations indicating any relationship between maximum spur length and the second longest bill (*n* = 19, *r* = .321, *p* = .169, spatially corrected *p* = .216), nor a relationship between maximum spur length and the third longest bill value (*n* = 15, *r* = .141, *p* = .604, spatially corrected *p* = .68).

## 4 DISCUSSION

In this study, we found that assemblage mean and maximum values of sunbird bill lengths were positively correlated with mean and maximum *Impatiens* spur lengths across locations. These positive correlations hold only for forest sunbird assemblages sharing the same habitat with *Impatiens* species. The long‐billed sunbirds seem to locally match the morphology of multiple *Impatiens* plant species, not vice versa. This suggests evidence for *trait matching* between taxa involved in pollination/nectarivory interactions at assemblage level and over a large spatial scale in tropical Africa, which has been never shown before. The geographical variation in trait‐matching generally remains poorly explicitly investigated, even though Sonne et al. (2019) recently showed that the trait‐matching within hummingbird–plant communities is influenced by spatial distribution of morphotypes. In our study, we showed that geographical space use of involved taxa determined by habitat selection contributes to the observed trait matching. As *Impatiens* plants inhabit mostly forest habitats (Grey‐Wilson, 1980), we performed the analyses for forest and nonforest sunbird species separately. In forest sunbirds, the trait correlation was significant for mean assemblage values of the traits as well as for the maximum values within assemblages. However, we found no significant correlation between the minimum trait values. This indicates that the correlation between mean assemblage trait values is likely to be driven by the relationship between long‐billed sunbirds and long‐spurred flowers within assemblages. The absence of any relationship between short‐billed sunbirds and short‐spurred *Impatiens* flowers fits the hypothesis that short‐billed birds and short‐spurred plants are nonspecialized members of the assemblages with low specificity, interacting with wide spectra of other plants/birds and creating low reciprocal selection pressure (Fenster, 1991; Maruyama et al., 2014).

In contrast to forest sunbirds, negative relationships were found for the nonforest group of sunbirds. These findings suggest that the positive relationship between the traits holds only for syntopical assemblages, as only forest sunbirds are in real touch with *Impatiens* plants growing in the forest undergrowth. From this point of view, we can support the idea of habitat specificity of trait matching (Maruyama et al., 2014; Stiles, 1981).

Our data suggest an existence of the correlation between traits of members of forest sunbird and *Impatiens* assemblages. Observed trait matching implies the existence of evolutionary induced adaptations driven by co‐evolutionary processes and/or by changes in *Impatiens* morphology due to pollinator shifts (Whittall & Hodges, 2007; see also Tripp & McDade, 2013), even though it is not a direct evidence for it. Based on the approach we employed, we just confirmed that sunbird and *Impatiens* species in local assemblages are combined nonrandomly in respect to bill/spur length. Apart from evolutionary processes, it might be a result of an environmental filter acting independently on both traits (Janzen, 1980; Nuismer et al., 2010). Nevertheless, we assume that this scenario is not very likely as it is difficult to imagine mechanism independent on plant–sunbird interactions, which selects in the same direction such different traits as are the bird bills and floral spurs. In fact, the selection pressure independent of pollination/nectarivory processes and responsible for prolongation of both traits is difficult to propose. However, the correlation at assemblage level comprises the fact that different species pairs contribute to overall pattern between locations. In other words, our results show that various long‐billed sunbirds are likely to be found sympatrically/syntopically with various longed spurred *Impatients* plants, even though specialized bird–plant pairs can be identified (see Table S1). As a result, a long‐billed sunbird can be geographically associated with different species of *Impatiens* plants, however most likely with long floral tubes. This indicates either (a) community assembly based on trait matching (i.e., ecological fitting, Janeček et al., 2020) or (b) community wide adaptations, that is co‐evolutionary relationships based on actual trait values in the community rather than strict species–species relationships, which is indicated also by our analyses of higher order correlations (see discussion below). As long‐billed sunbirds co‐occur with different *Impatiens* species, the last scenario that sunbirds/*Impatiens* species prolong their bills/spurs during interactions with different species during their evolutionary history seems to be more likely.

In this context of specialization in species interactions, we tested for specialization asymmetry in the *Impatiens*–sunbird pollination system. Our analyses revealed that the bill values of birds with the longest bills in the assemblage tend to correlate also with lower‐order *Impatiens* species in terms of spur length (the 3rd one in particular). However, we found no evidence for the opposite situation (i.e., a correlation between spur values of *Impatiens* plants with the longest spur and lower‐order bill lengths). This finding is in accordance with the asymmetric interactions commonly described in plant–pollinator assemblages (Vázquez & Aizen, 2004) and with our recent observations on Mt. Cameroon where all *Impatiens* species are pollinated just by one long‐billed sunbird (Bartoš & Janeček, 2014; Janeček et al., 2015). Besides, it indicates that a scenario of one sunbird species matching the traits of several *Impatiens* species at a particular location is more likely than the reverse situation. A similar pattern has been described by Geerts and Pauw (2009) in South Africa. They showed that a high number of specialized plants are related with one long‐billed sunbird, *Nectarinia famosa*. Alternatively, the observation that one sunbird species matches the traits of several plant species may show that *Impatiens* species tend to be more specialized for sunbirds than vice versa. This agrees with the idea that whereas long corolla tubes are highly ecologically specialized (Fenster, 1991), long bills represent a general adaptation because they enable birds to exploit a large spectrum of flowers (Cotton, 1998). We also agree with the opinion that although pairwise selection is rare, selection is often driven by a small number of “driving” species (Temeles et al., 2009).

Looking at species representing maximum bill size values in our dataset (see Table S1), the genus *Cyanomitra* is frequently present. The close relationship between *Impatiens* plants and sunbirds has already been demonstrated at the local scale: Janeček et al. (2011, 2012) have shown that sunbirds (especially *Cyanomitra oritis*) are important pollinators of *Impatiens sakeriana*. In addition, a strong bond between these two species is suggested by specific morphological and behavioral adaptations (Janeček et al., 2011, 2012). Although there is unfortunately not enough published information about other members of the focal taxonomic groups, some preliminary data indicate tight relationships also between other species of the genus *Impatiens* and sunbirds (Janeček et al., 2015). Based on our field experience from Afrotropical forests, such a close relationship is not surprising. *Cyanomitra* sunbirds search for food preferentially in the forest undergrowth (which distinguishes them from other sunbird species), where *Impatiens* plants are an abundant source of nectar. The probability of their mutual interaction is thus relatively high. In addition, breeding activity of *Cyanomitra oritis* seems to be shifted compared to other syntopically occurring sunbirds and synchronized with flowering of *Impatiens* species in the Cameroon Mts. (Fotso, 1996; Hořák et al., unpublished results). It is worth noting that the existence of such a highly specialized plant–bird pollination system within the forest environment resembles the situation already described in the New World. In South America, highly specialized ornithophilous undergrowth herbs of the genus *Heliconia* are reported to have high affinity to small number of long‐billed hermit hummingbirds (Dalsgaard et al., 2009; Stiles, 1975, 1981). Similarly, the most morphologically specialized plants are pollinated by one hermit *Phaetornis pretrei* in forest formations in Neotropical savanna (Maruyama et al., 2014) and many species of *Passiflora* with long nectar tubes are dependent on long‐billed hummingbird *Ensifera ensifera* in Andes (Abrahamczyk et al., 2014).

Although our study represents the first large‐scale test of a sunbird–plant trait matching hypothesis in tropical Africa, it has limitations, and considerable amount of work should be done to confirm or refute our conclusions. Intensive field studies on the organization of nectarivorous birds and related plant assemblages are particularly required. These are commonly available for better explored regions (e.g., Dalsgaard et al., 2009; Feinsinger, 1976; Feinsinger & Colwell, 1978; Wolf et al., 1976), however, very rare in tropical Africa (Gill & Conway, 1979; Janeček et al., 2012). This is in fact the only way by which we can describe real relationships between individual sunbird and *Impatiens* species.

In conclusion, our observation of a phenotypic correlation between flower spur lengths and bird bill lengths over large geographical scale and across different species suggests highly specialized pollination interactions between *Impatiens* species and sunbirds in the mountains of tropical Africa. Based on the system studied, we propose that special habitat preferences (e.g., for forest undergrowth) of interacting organisms might locally lead to co‐existence of highly specialized nectar‐feeding bird and nectar producing plant species. This, together with geographical isolation of montane environments, might increase the spatial–temporal probability of mutual interactions between the species and thus enhance the strength of their relationship. However, our understanding of plant–sunbird co‐existence in tropical Africa is still very shallow, and conclusions made here should be treated with caution. They are based on a large‐scale pattern of average species' traits, and follow‐up field work is needed to provide a more realistic picture of geographical variation in morphological and behavioral co‐adaptations between *Impatiens* species and sunbirds in African montane forests.

## CONFLICT OF INTEREST

None declared.

## AUTHOR CONTRIBUTIONS

**David Hořák:** Conceptualization (equal); Data curation (equal); Formal analysis (equal); Funding acquisition (equal); Investigation (equal); Methodology (equal); Project administration (equal); Resources (equal); Software (equal); Supervision (equal); Validation (equal); Visualization (equal); Writing‐original draft (equal); Writing‐review & editing (equal). **Štěpán Janeček:** Conceptualization (equal); Data curation (equal); Formal analysis (equal); Funding acquisition (equal); Investigation (equal); Methodology (equal); Project administration (equal); Resources (equal); Software (equal); Supervision (equal); Validation (equal); Visualization (equal); Writing‐original draft (equal); Writing‐review & editing (equal).

## Supporting information

Table S1

## Data Availability

Data are deposited on datadryad.org, https://doi.org/10.5061/dryad.gxd2547k7.
