# Supplementary material for: A geographical perspective on the relationship between Impatiens spur lengths and bill lengths of sunbirds in Afrotropical mountains
Source: Ecol Evol. 2021 Feb 10;11(7):3120–9. doi: 10.1002/ece3.7258 (PMC8019056; doi:10.1002/ece3.7258)
Supplement: Supplementary file 1 — Table S1 [file ECE3-11-3120-s001.docx]

Table S1. Locations of studied assemblages on mountains and islands in tropical Africa. Descriptive statistics of target morphological traits of *Impatietns* plants and sunbird assemblages are provided. For maximum values of traits within assemblages, the species scientific name is given.

| Location | Country | Position^1^ | | Spur length (mm)^2^ | | | Bill length (mm)^3^ | | | | | |
| --- | --- | --- | --- | --- | --- | --- | --- | --- | --- | --- | --- | --- |
|  |  |  | |  | | | Forest species | | | Non-Forest species | | |
|  |  | Latitude | Longitude | Max | Mean | Min | Max | Mean | Min | Max | Mean | Min |
| Sao Tomé | Sao Tomé and Principe | 0.18 | 6.58 | 32.37  *I. buccinalis* | 32.37 | 32.37 | 36.75  *Dreptes thomensis* | 26.98 | 17.20 | - | - | - |
| Principe | Sao Tomé and Principe | 1.67 | 7.40 | 21.36  *I. manteorana* | 21.36 | 21.36 | 26.05  *Cyanomitra obscura obscura* | 23.90 | 21.75 | - | - | - |
| Bioko | Equatorial Guinea | 3.62 | 8.75 | 30.32  *I. hians var. hians* | 21.40 | 14.80 | 27.40  *Cyanomitra oritis poensis* | 19.08 | 14.10 | 21.15 | 20.08 | 19.00 |
| Mt. Cameroon | Cameroon | 4.20 | 9.17 | 30.32  *I. hians var. hians* | 22.30 | 14.80 | 35.30  *Cinnyris superba superba* | 20.39 | 14.10 | 26.65 | 21.58 | 14.20 |
| Cameroon Mts. | Cameroon | 6.06 | 10.12 | 30.32  *I. hians var. hians* | 23.27 | 14.80 | 35.30  *Cinnyris superba superba* | 21.16 | 14.10 | 27.40 | 20.99 | 12.00 |
| Crystal Mts. | Gabon | -0.77 | 10.22 | 30.32  *I. hians var. hians* | 25.87 | 19.08 | 35.30  *Cinnyris superba superba* | 20.11 | 14.10 | 21.15 | 18.12 | 14.20 |
| Massif du Chailu | Gabon | -2.53 | 11.17 | 30.32  *I. hians var. hians* | 23.62 | 17.50 | 26.05  *Cyanomitra obscura cephaelis* | 18.28 | 14.10 | 21.15 | 19.02 | 16.90 |
| Ethiopian Highlands | Ethiopia | 9.93 | 38.15 | 16.66  I. rothii | 16.66 | 16.66 | - | - | - | 31.10 | 24.88 | 17.45 |
| Mt. Elgon | Uganda / Kenya | 1.08 | 34.48 | 28.60  *I. digitata ssp. phlictidoceras* | 19.82 | 11.77 | 26.05  *Cyanomitra obscura vencenti* | 17.48 | 14.10 | 31.10 | 22.72 | 16.15 |
|  |  |  |  |  |  |  |  |  |  |  |  |  |
| Mt. Kenya | Kenya | -0.17 | 37.37 | 18.71  *I. fischeri* | 18.71 | 18.71 | 27.90  *Cyanomitra olivacea neglecta* | 19.34 | 14.35 | 34.30 | 26.49 | 17.45 |
| Aberdare Mts. | Kenya | -0.62 | 36.70 | 18.71  *I. fischeri* | 18.71 | 18.71 | 27.90  *Cyanomitra olivacea neglecta* | 19.34 | 14.35 | 34.30 | 26.49 | 17.45 |
|  |  |  |  |  |  |  |  |  |  |  |  |  |
| Mt. Kilimanjaro | Tanzania | -3.07 | 37.33 | 21.09  *I. digitata ssp. digitata* | 16.40 | 13.44 | 27.90  *Cyanomitra olivacea neglecta* | 21.00 | 16.60 | 34.30 | 26.53 | 17.45 |
|  |  |  |  |  |  |  |  |  |  |  |  |  |
| Ngorongoro | Tanzania | -3.23 | 35.48 | 15.14  *I. digitata ssp. jageri* | 15.14 | 15.14 | - |  |  | 27.50 | 23.15 | 17.45 |
|  |  |  |  |  |  |  |  |  |  |  |  |  |
|  |  |  |  |  |  |  |  |  |  |  |  |  |
| Nguru Mts. | Tanzania | -5.98 | 37.50 | 26.70  *I. nguruensis* | 21.62 | 16.53 | 27.90  *Cyanomitra olivacea neglecta* | 19.67 | 13.75 | 27.80 | 21.98 | 16.90 |
| Ukaguru Mts. | Tanzania | -6.47 | 36.83 | 23.31  *I. ukagurensis* | 23.31 | 23.31 | 27.90  *Cyanomitra olivacea neglecta* | 21.15 | 16.60 | 30.30 | 22.39 | 16.90 |
| Uluguru Mts. | Tanzania | -7.23 | 37.55 | 19.34  *I. tricaudata* | 16.44 | 13.45 | 27.90  *Cyanomitra olivacea neglecta* | 20.43 | 13.75 | 27.80 | 21.92 | 16.90 |
|  |  |  |  |  |  |  |  |  |  |  |  |  |
| Udzungwa Mts. | Tanzania | -7.78 | 36.65 | 16.53  *I. keilii* | 16.53 | 16.53 | 27.90  *Cyanomitra olivacea neglecta* | 19.91 | 13.75 | 30.30 | 20.78 | 13.10 |
| Mahange | Tanzania | -4.90 | 38.45 | 16.53  *I. keilii* | 16.53 | 16.53 | 18.50  *Cinnyris mediocris mediocris* | 17.55 | 16.60 | 31.10 | 23.61 | 16.15 |
|  |  |  |  |  |  |  |  |  |  |  |  |  |
|  |  |  |  |  |  |  |  |  |  |  |  |  |
| Chimanimani Mts. | Mozambique | -15.95 | 35.58 | 13.00  *I. salpinx* | 13.00 | 13.00 | 26.05  *Cyanomitra obscura scleteri* | 21.33 | 16.60 | 33.65 | 23.04 | 16.90 |
| Rift 1 | Tanzania | -8.18 | 31.45 | 17.64  *I. austrotanzanica* | 15.90 | 14.15 | 17.60  *Hedydipna collaris garguensis* | 17.60 | 17.60 | 30.30 | 22.29 | 16.15 |
|  |  |  |  |  |  |  |  |  |  |  |  |  |
| Rift 2 | DRC | -3.68 | 28.12 | 19.08  *I. niamniamensis* | 15.02 | 10.96 | 31.25  *Cinnyris johannae* | 20.78 | 14.35 | 30.30 | 23.05 | 16.90 |
| Rift 3 | DRC | -0.93 | 28.87 | 24.0  *I. pierlotii* | 17.17 | 10.96 | 35.30  *Cinnyris superba buvuma* | 21.69 | 14.10 | 30.30 | 22.68 | 17.45 |
| Rift 4 | DRC | 2.00 | 30.52 | 19.08  *I. niamniamensis* | 19.08 | 19.08 | 29.25  *Cyanomitra alinae alinae* | 20.67 | 14.10 | 30.30 | 20.75 | 12.00 |
| Rift 5 | Burundi / Rwanda / Uganda | -1.88 | 30.55 | 25.00  *I. nyungwensis* | 18.09 | 10.96 | 35.30  *Cyanomitra superba buvuma* | 22.89 | 14.10 | 30.30 | 21.83 | 16.90 |
| Ruwenzori | Uganda / DRC | -0.10 | 30.07 | 19.08  *I. niamniamensis* | 15.41 | 11.33 | 29.25  *Cyanomitra alinae alinae* | 19.51 | 14.10 | 28.55 | 22.03 | 17.45 |

^1^ Geographical position of particular study locations

^2^ Length of the spur of *Impatiens* flowers in mm

^3^ Length of the bill of sunbird species in mm, separately for species inhabiting forest and non-forest habitats
